# Reusability of Discarded Tubular Ceramic Membranes for CO_2_ Removal: A Case Study for Membrane Circularity

**DOI:** 10.1021/acsomega.3c02568

**Published:** 2023-07-31

**Authors:** Elcim Karatas, Sama A. Al-Mutwalli, Mustafa N. Taher, Mohammad Mahdi A. Shirazi, Derya Y. Koseoglu-Imer

**Affiliations:** † Department of Environmental Engineering, Istanbul Technical University, Istanbul 34467, Turkey; ‡ Membrane Industry Development Institute, Tehran 1587-856614, Iran

## Abstract

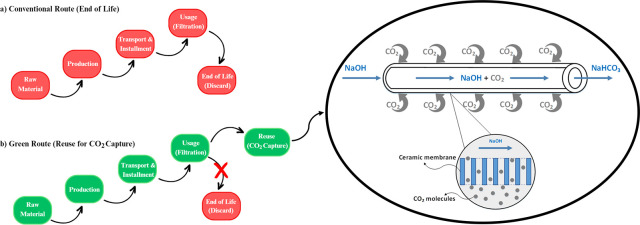

Discarded polymeric or ceramic membranes are currently in need of appropriate and sustainable management. In the present study, the direct reuse of discarded ceramic membranes in membrane contactor (MC) systems for CO_2_ removal was investigated for the first time. The hydrophobic surface modification of the discarded ceramic membrane was done by using macromolecule additive coating. The influence of operational parameters (absorbent liquid flow rate (*Q*_L_), feed gas flow rate (*Q*_g_), and different NaOH concentrations) of the MC on CO_2_ removal was investigated to prove the technical feasibility of reused ceramic membranes. The CO_2_ absorption flux was 7.9 × 10^–4^ mol/m^2^ s at optimal conditions of 2 M NaOH, *Q*_L_ (20 mL/min), and *Q*_g_ (300 mL/min) with a removal efficiency of 98%, which lasted for 8 h. This study demonstrates a potential alternative for the reuse of discarded ceramic membranes and avoids their disposal in landfills. The proposed approach will also bring membrane technology into the circular economy and achieve sustainability goals by reducing the amount of waste from discarded ceramic membranes in the future and combating global warming by absorbing CO_2_.

## 1 Introduction

Recently, the issues of global warming and sustainability have come to the fore. On the one hand, sustainability is about the economical use of natural resources and the search for environmentally friendly alternatives to these resources. On the other hand, global warming is mainly about greenhouse gas (GHG) emissions, which are increasing due to human activities in modern times.^1,2^ At the top of the list of GHGs is carbon dioxide (CO_2_), which is considered one of the main contributors to global warming and climate change.^3,4^ In the last 30 years, CO_2_ emissions into the atmosphere from various sources have increased by almost 55% and are predicted to increase by 90% by 2050.^5^ Regarding CO_2_ in an indoor environment, recent studies have shown that high indoor CO_2_ levels can have negative effects on human health.^6^ Other studies suggest that high CO_2_ levels are a potential transmission risk for respiratory infectious diseases such as COVID-19.^7^ Moreover, the removal technology of CO_2_ from the atmosphere and indoor air has become a major concern in recent years. Therefore, the implementation of reliable and cost-effective technologies to remove CO_2_ from outdoor and indoor air is an important concern.

One of the much-discussed methods is the absorption of CO_2_ using various absorbents. Despite the success of this method, its application involves high initial investment and operating costs as well as technological disadvantages such as energy consumption, cost and energy of regeneration, and corrosion problems in the plant facilities.^8^ The gas–liquid membrane contactor (MC) provides a suitable technological alternative for the removal of CO_2_, as it combines the membrane separation and the absorption process to overcome the obstacles observed when using the absorption process individually.^9^ MC systems offer a large interfacial area, more flexible and controlled operating conditions for gas (*Q*_g_) and liquid (*Q*_L_) flow rates, no emulsion formation, no discharge and flooding at different flow rates, and no difference in density between the liquids involved in the process, an easy scale-up procedure, as well as compactness and energy saving.^9,10^ Polymeric membranes are mainly used in MC systems, while other types of membranes, such as ceramic membranes, have not been extensively researched yet. Ceramic membranes can be an excellent candidate for use in MCs due to their superior chemical stability due to the hydrophobic coating, temperature resistance, corrosion resistance, and mechanical strength.^11^

Membranes are widely used for water reclamation and wastewater treatment. Advances in membrane technology require a more holistic and sustainable approach to membrane processes.^12^ In particular, the identification of secondary uses of membranes that become waste after primary use without being disposed of. This can be a sustainable approach to reduce the waste that is generated when these membranes are disposed of in the environment. Considering their average lifetime, their waste loads are very high. Consequently, it is very important to consider the recycling and reuse of discarded membranes.^13,14^ Recently, studies have focused on the reusability of used polymeric membranes or their conversion into another type of membrane. A good example is the conversion of used reverse osmosis membranes into ultrafiltration (UF) or nanofiltration membranes by chemical treatment.^15−18^ However, this conversion method uses aggressive chemicals that are considered harmful to the environment. In addition to polymeric membranes, ceramic membranes have also been widely used in water and wastewater treatment in recent years.^19^ The first large-scale installation of ceramic membranes was in Japan in 1998.^20^ It is estimated that the average annual growth rate of the ceramic membrane market is 11.3%, and the market size is growing rapidly with an incremental growth of USD 3.1 billion between 2021 and 2025.^21^ The lifetime of polymeric membranes should be at least 6 years, while the lifetime of ceramic membranes is reported to be more than 20 years.^22^ To the best of our knowledge, the reusability of discarded ceramic membranes has not been studied yet. In particular, few studies were found on the reuse or recycling of ceramic membranes in filtration applications. For example, a patent by Wang et al.^23^ describes a combination of heat treatment and chemical cleaning for the reuse of ceramic filters. This indicates that there is no systematic approach to dealing with ceramic membranes after their lifetime.

The aim of this study is to investigate the reusability of tubular ceramic membranes (TCM) that have been used for the treatment of industrial wastewater. The TCM was reused in a MC system for the capture of CO_2_ from indoor air. The operational parameters (*Q*_g_, *Q*_L_, and the effect of different absorption solution concentrations) were investigated, and an approach for managing reusability was reported. The successful implementation of this MC system with TCM represents a circular economy approach for ceramic membranes at the end of their lifetime.

## 2 Fundamentals of Involved Processes

### 2.1 CO_2_ Absorption by NaOH

CO_2_ is absorbed by NaOH according to the chemical reactions shown below. When CO_2_ is directed to an aqueous solution, it tends to be physically absorbed into the aqueous CO_2_, as shown in eq 1. When NaOH is used to absorb CO_2_, it is completely ionized in water to form Na^+^ and OH^–^.^24,25^ When CO_2_ comes into contact with NaOH solution, it reacts with OH^–^ to produce HCO_3_^–^ and CO_3_^2–^, according to eqs 2 and 3. Due to the presence of OH^–^, aqueous CO_2_ is rapidly consumed according to eqs 2 and 3 as soon as it is formed.^24,26^ In the early stages of the absorption process, eq 3 is predominant, causing the CO_3_ concentration to increase to the HCO_3_^–^ concentration at high alkalinity values. As a result of eqs 2 and 3, OH^–^ decreases rapidly, leading to a decrease in pH at the early stages of the absorption process. Equation 4 represents the overall reaction in the early stages of CO_2_ absorption by NaOH. The absorption of CO_2_ by the NaOH solution continues when CO_2_ is added. This is followed by the consumption of OH^–^ in conjunction with a decrease in pH, which increases CO_3_^2–^ accumulation, which in turn increases the reverse reaction of eq 3 and the forward reaction of eq 2 simultaneously. The overall absorption reaction in aqueous solution can be formulated as shown in eq 5. Yoo et al.^24^ pointed out that when the absorption equilibrium is reached, a small absorption of CO_2_ is possible, which balances the unabsorbed CO_2_:
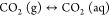
1
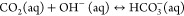
2
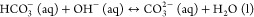
3
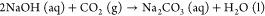
4
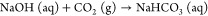
5

### 2.2 Membrane Contactors

**Figure 1:**
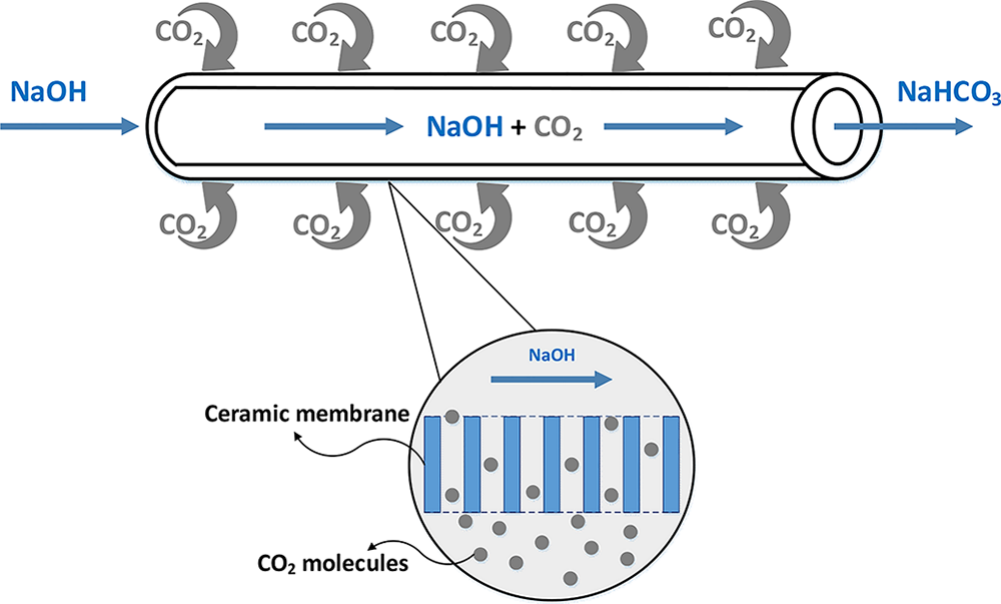
Mechanism of membrane contactor for absorption of CO_2_.

The main difference between MC systems and other membrane processes is that porous membranes are used as carriers to bring two phases into contact and allow mass transfer between them. MCs combine two different processes, chemical absorption and membrane separation, in one system.^4^ The driving force of the liquid absorbent in MCs depends on the concentration difference. The CO_2_ is separated from the gas phase along the membrane surface and transferred to the liquid absorbent. Usually, hydrophobic membranes are used in MC systems to limit the flux of the absorbent liquid to the membrane surface and thus prevent it from entering the pores. It is important to provide a hydrophobic surface in MCs to maintain interference between the gas and liquid phases. As a result, CO_2_ mass transfer and separation efficiency are improved. Figure 1 shows the mechanism of the MC system in CO_2_ absorption used in this study, which works with a counterflow between gas and liquid. Once the gas flows through the system, the pores are filled with gas molecules, and then the gas diffuses through the membrane pores and comes into contact with the NaOH solution (absorbent) on the inner ceramic membrane surface. Once the CO_2_ comes into contact with the NaOH solution, absorption reactions occur according to the equations mentioned in the previous section. Operating MC systems in this way helps to avoid the obstacles observed in conventional CO_2_ absorption systems and allows separate control of the liquid and gas flow. In addition, MCs achieve exceptionally high CO_2_ mass transfer rates due to the greater surface area per unit volume that they provide compared to conventional methods.^3,4^ In addition, gas and liquid can be controlled independently to prevent overflow, bubble formation, etc. By controlling the operating problems, *Q*_g_ can be adjusted and CO_2_ absorption capacity can be increased for the same unit volume. MC provides about 30 times more effective surface area than the conventional absorption process, and the literature reports that the size of the absorption process can be reduced by 10 times.^27^ These are systems where scale-up is advantageous as the surface area can be easily controlled by the number of membrane modules per unit, and the system is relatively easy to operate.

## 3 Materials and Methods

### 3.1 Materials

Phosphoric acid 85% (H_3_PO_4_), sodium acetate trihydrate (C_2_H_9_NaO_5_), and sodium carbonate (Na_2_CO_3_) were purchased from Merck Millipore (USA). Methyltrichlorosilane (MTS) was purchased from Sigma-Aldrich. Sodium hydroxide (NaOH) was purchased from Fisher Scientific, England. The TCM (15 kDa) was purchased from Sterlitech Corporation, USA. According to the supplier, the active and support layers of the TCM are made of zirconia (ZrO_2_) and titanium oxide (TiO_2_), respectively. The TCM used has an outer diameter of 1.0 cm, an inner diameter of 0.6 cm, a length of 25 cm, and a maximum filtration area of 40 cm^2^. The TCM used in this study has already been used in a laboratory-scale crossflow module for whey filtration. The TCM was used for 2 years and lost its original performance by 30–35% as irreversible fouling. Before using the TCM, chemical membrane cleaning was performed in accordance with the procedure found elsewhere.^28^ The cleaning was performed in two stages: first, the TCM was dipped in 0.4 N NaOH for 30 min at 85 °C to remove the organic foulants, followed by a dip in distilled water to neutralize the pH. Then, TCM was dipped in 0.04 N H_3_PO_4_ for 15 min at 50 °C to remove inorganic foulants, followed by a dip in distilled water to neutralize the pH. Following the chemical cleaning, the water flux was determined using distilled water.

### 3.2 Hydrophobic Coating Procedure of TCM

To reduce the presence of hydroxyl groups, the TCM was hydrophobically coated with 7% MTS after chemical cleaning steps. Prior to coating, the membrane module was dehumidified for 24 h and dried in an oven at 100 °C. After the membrane cooled to ambient temperature, it was immersed and stored for 24 h in 30 mL of a 7% MTS solution mixed with ethyl acetate. The hydrophobic module was then washed with pure ethyl acetate, dried, and used for the experiments. The hydrophobic coating was used in the experiments after many areas of the module had been inspected. The contact angle values of membranes were measured with a tensiometer from KSV Instruments before and after coating. A drop of distilled water was applied to the e surface using a stainless steel syringe needle. Ten measurements were done at different locations on the membrane surface and averaged to determine the mean contact angle. The contact angle of discarded ceramic membrane increased from 48.0 ± 7.0 to 120.0 ± 6.0° after coating, indicating a more hydrophobic surface.

### 3.3 Experimental Setup

**Figure 2:**
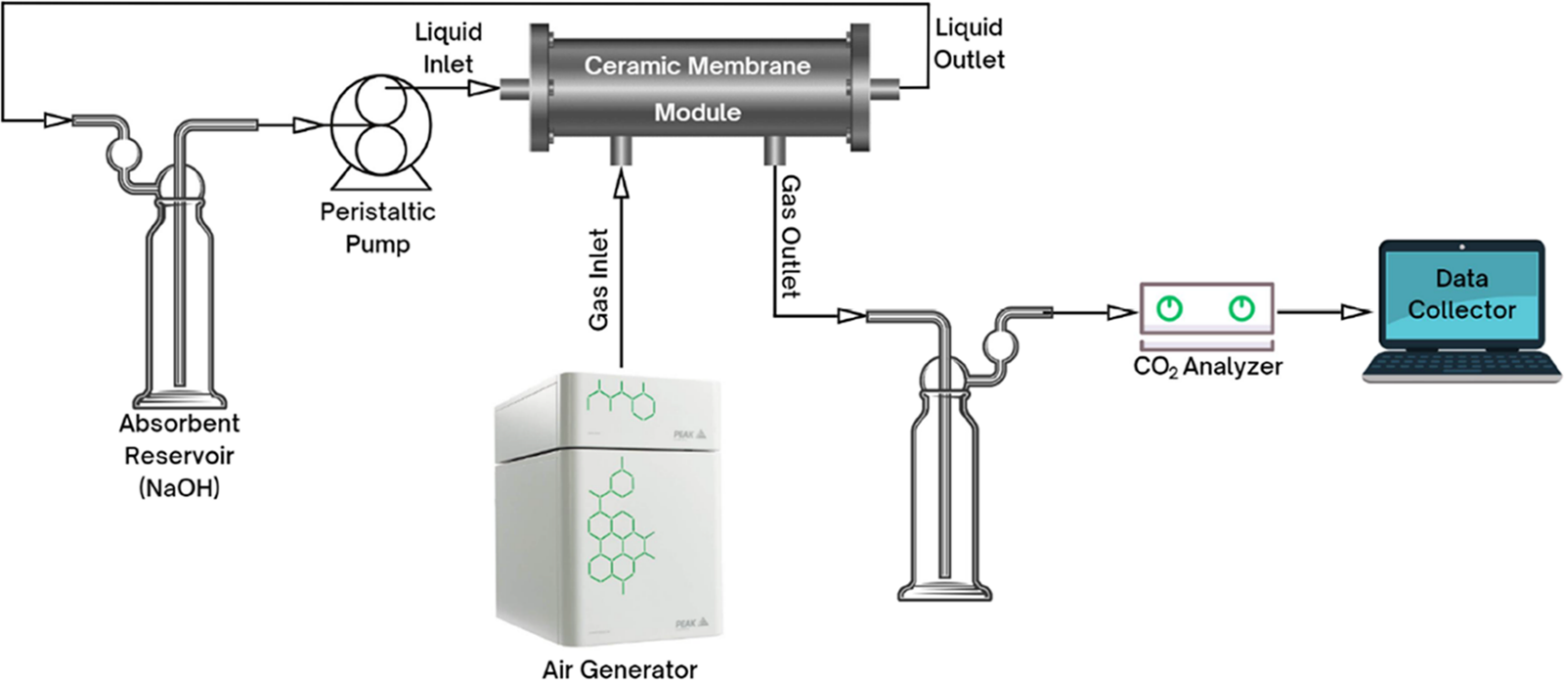
Schematic representation of the laboratory scale tubular ceramic MC system.

To test the removal of CO_2_ from indoor air using the TCM process, a laboratory-scale MC was installed. The TCM was aligned horizontally inside the MC model, as shown in Figure 2. The absorbent solution was pumped into the MC model through the inside of the TCM using a peristaltic pump (Gear Drive Pump, Cole-Parmer) in a countercurrent mode, i.e., liquid through the lumen of TCM and gas through the shell surface of the TCM. The indoor air was supplied to the MC at an adjustable constant flow rate through an air generator (Peak-Air generator, UK) with a deactivated CO_2_ removal system and a mass flow meter. The amount of CO_2_ in the outlet gas was measured in ppm once per second throughout the experiment using a CO_2_ gas analysis system (TRL Instruments, Turkey). Inlet and outlet *Q*_g_ were continuously monitored with a flow meter to avoid any leakage. At the beginning of each test, the hydrophobicity of the membrane was checked to ensure that the membrane did not get wet, and the silane coating of the membranes was renewed as needed.

The CO_2_ removal efficiency (η) is one of the most accurate indicators for evaluating the performance of MC, which can be calculated according to eq 6:^29^
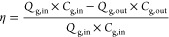
6where η is the CO_2_ removal efficiency (%), *C*_g,in_ and *C*_g,out_ are CO_2_ volumetric fraction (%) in feed and outlet gas, respectively, and *Q*_g,in_ and *Q*_g,out_ are flow rate (m^3^/h) of feed gas and outlet gas, respectively. CO_2_ mass transfer flux (*J*_CO_2__) is another important parameter and was calculated by eq 7:^30^
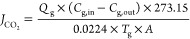
7where *J*_CO_2__ is the CO_2_ mass transfer flux (mol/m^2^ s), *Q*_g_ is the gas flow rate in MC (m^3^/h), *C*_g,in_ and *C*_g,out_ are CO_2_ molar concentrations (mol/m^3^) in feed and outlet gas, respectively, *T*_g_ is temperature value (K), and *A* is the contact area of gas and liquid (m^2^).

### 3.4 Effect of Different Operation Parameters

MC systems are affected by different parameters such as *Q*_L_ and *Q*_g_, the concentration of absorption solution, CO_2_ concentration, liquid temperature, gas inlet pressure, and liquid inlet pressure.^31^ In this study, the operating parameters investigated are *Q*_L_, *Q*_g_, and the concentration of NaOH in the absorption solution. The system shown in Figure 2 was operated at different *Q*_L_ values of 20, 60, 80, and 100 mL/min. In this phase of the study, 2 M NaOH solution was circulated in the MC system at a *Q*_g_ of 300 mL/min for an observation period of 8 h. In the following phase, the system was operated at a *Q*_g_ of 300, 600, and 900 mL/min at a fixed *Q*_L_ for a similar observation period. Different *Q*_L_ and *Q*_g_ were investigated to ensure that the gas did not come into direct contact with the liquid phase, bubbles did not form in the absorbent solution, and liquid did not enter the gas stream. In the final phase of this study, the effect of different concentrations of absorbent solution was investigated. The NaOH concentrations used were 1.0, 1.5, and 2.0 M NaOH. The optimization of this parameter is for economic consideration of the cost of the chemicals used for CO_2_ absorption. All experiments in the current study were conducted at ambient temperature. All values reported in this study reflect the average values of the experiments performed in triplicate at each step.

## 4 Results and Discussion

### 4.1 Membrane Cleaning before Reuse

**Figure 3:**
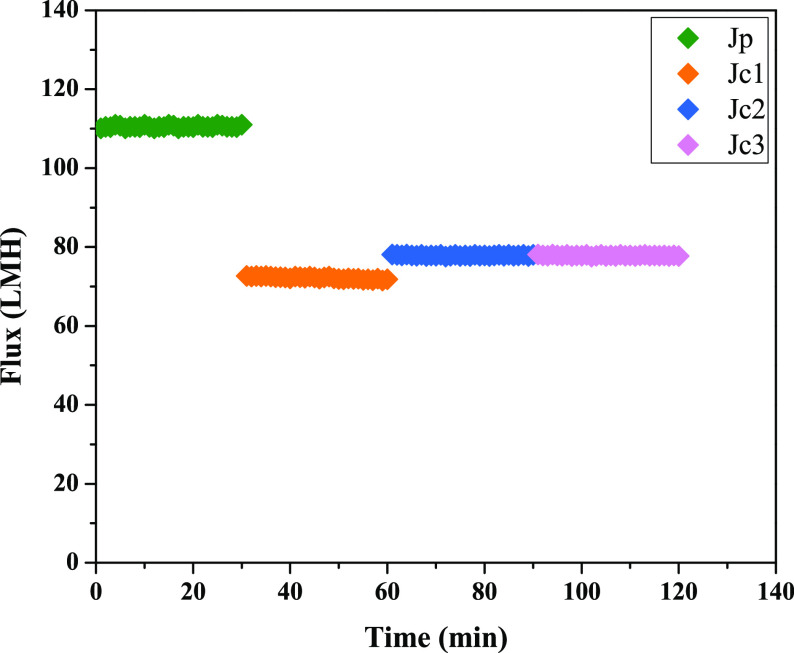
Water flux of pristine and discarded chemically cleaned TCM.

Before coating, the discarded TCM was chemically cleaned to remove foulants from its structure. Prior to this, the pure water flux (*J*_p_) of the TCM was determined by filtering distilled water at 2 bar for 30 min and found as 110 L/m^2^ h, as shown in Figure 3 with green color. After the membrane had reached the end of its useful life, the TCM was cleaned as described in Section 3.1, and the flux after cleaning was determined in a similar way to *J*_p_. After the first cleaning, the flux (*J*_c1_) was about 71.1 L/m^2^ h, indicating a flux recovery rate of 65%. Subsequently, the TCM was cleaned two more times using the same cleaning procedure as mentioned earlier. The flux results showed that the flux recovery rate increased to about 71%, with a flux (*J*_c2_ and *J*_c3_) value of 78.4 L/m^2^ h. The flux values shown in Figure 3 indicate that the TCM is subject to irreversible fouling, where the foulants cannot be removed from the membrane, resulting in a decrease in its original performance. Consequently, this membrane cannot be recommended for further use for water/wastewater filtration. Normally, after the ceramic membrane reaches the end of its lifespan, it is disposed of in a landfill. However, this work proposes another way in which discarded TCMs can be reused in MC systems for capturing CO_2_ from indoor air.

### 4.2 Effect of Absorbent Flow Rate on CO_2_ Removal Efficiency

**Figure 4:**
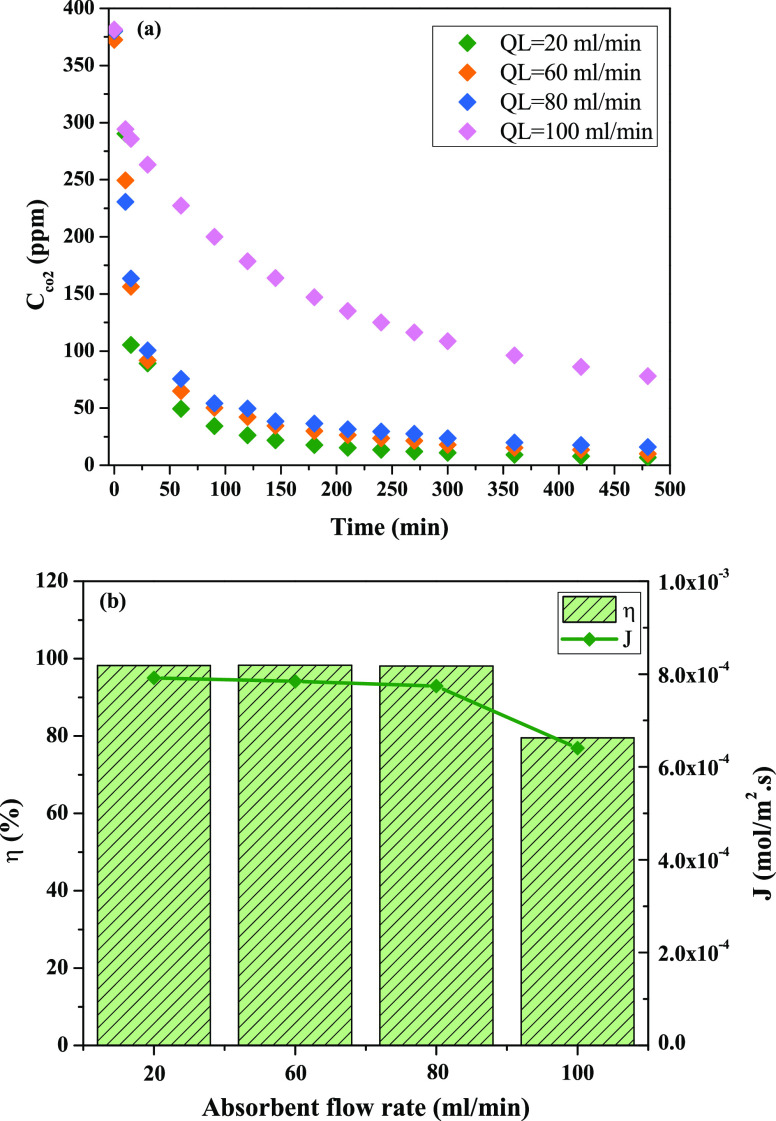
(a) Time-dependent variation of CO_2_ concentration in outlet gas flow for different absorbent flow rates, and (b) effects of absorbent flow rate (*Q*_L_) on CO_2_ removal (worked at *Q*_g_ = 300 mL/min and NaOH concentration = 2 M).

The flow rate of absorbent (*Q*_L_) and the resistance of the liquid phase are important operating parameters affecting the mass transfer of physical absorption in MC systems.^32^ In the first phase of the experiments, the effect of 2 M NaOH solution *Q*_L_ on CO_2_ removal was investigated by circulating 20, 60, 80, and 100 mL/min at a constant *Q*_g_ of 300 mL/min for 8 h in the system. It was assumed that the pores of the membrane were filled with gas in the “non-wetting mode”. During the process, the gas diffuses on the outer surface of the membrane and penetrates through the pores to the inner surface of the membrane. The gas then dissolves, diffuses, and undergoes chemical reactions with the absorbent. As can be seen in Figure 4a, the initial CO_2_ values for all experiments start at 380 ppm, which is a normal value for indoor air and decrease over time. The values in Figure 4a reflect the average results of experiments performed in triplicate at each flow rate of the absorbent. The *J*_CO_2__ and η values are shown in Figure 4b as a function of the flow rate of the absorbent. It was observed that when the flow rate of the absorbent was more than 100 mL/min, no flux could be obtained in the ceramic membrane because liquid droplets quickly appeared on the gas side after the system was operated. It was also observed that after 300 min, at a flow rate of absorbent of 100 mL/min, some liquid droplets formed on the gas side, so that η decreased at 100 mL/min.

It has been discussed in the literature that increasing *Q*_L_ decreases the resistance in the boundary layer that forms on the membrane surface and consequently increases the CO_2_ flux.^33^ In this study, η values were similar at *Q*_L_ values of 20, 60, and 80 mL/min with 98%, while the flux values were 7.92 × 10^–4^, 7.85 × 10^–4^, and 7.74 × 10^–4^ mol/m^2^ s, respectively. When the *Q*_L_ was increased to 100 mL/min, the η value decreased to almost 80% with a *J*_CO_2__ value of 6.40 × 10^–4^ mol/m^2^ s.

Although CO_2_ removal at liquid circulation rates of 60 and 80 mL/min is similar to that at 20 mL/min, the best η values in this experiment were obtained at a *Q*_L_ of 20 mL/min. This is in good agreement with the results reported by Lee et al.^34^ The authors introduced a modified hydrophobic Al_2_O_3_ hollow fiber membrane that could achieve a CO_2_ absorption flux of 7.8 × 10^–4^ mol/m^2^ s at an absorbent flow rate of 20 mL/min in a gas mixture of 20:80 vol % CO_2_:N_2_ at ambient conditions. In another work, Mansourizadeh and Ismail^35^ fabricated a polyvinylidene fluoride (PVDF)/glycerol mixed hollow fiber membrane and used water as an absorbent. The authors reported a CO_2_ flux of 7.9 × 10^–4^ mol/m^2^ s at an absorbent flux of 280 mL/min.

The results of the first part of this study suggest that a laboratory-scale gas–liquid MC using a discarded UF ceramic membrane can be successfully used to capture CO_2_ from indoor air using NaOH solution at ambient temperature. As shown in Table 1, the results obtained are very similar to those obtained in other studies under the same conditions.

**Table 1:** Comparison of Membrane Contactor Performance in Terms of CO_2_ Removal Efficiency and Flux^a^

membrane material	membrane module	CO_2_ percentage/concentration	absorbent solution (concentration)	liquid flow rate	gas flow rate	CO_2_ removal efficiency	CO_2_ flux (mol/m^2^ s)	reference
PVDF + surface modifying macromolecule	hollow fiber	100%	distilled water	50 (mL/min)			1.0 × 10^–3^	(32)
				300 (mL/min)			5.4 × 10^–3^	
ceramic membrane (Kaolin & Al_2_O_3_)	hollow fiber	100%	distilled water	20 (mL/min)		98%	7.92 × 10^–4^	(33)
				80 (mL/min)			7.74 × 10^–4^	
ceramic membrane (Al_2_O_3_)	hollow fiber	20%	ultrapure water	20 (mL/min)	50 (mL/min)		7.88 × 10^–3^	(34)
PVDF/glycerol	hollow fiber	100%	distilled water	280 (mL/min)	100 (mL/min)		7.9 × 10^–4^	(35)
ceramic membrane (Al_2_O_3_/ZrO_2_)	tube	12.5%	MEA (5%)	10 (mL/min)	50 (mL/min)	99%	7.5 × 10^–2^	(29)
					250 (mL/min)	51%	19.5 × 10^–2^	
ceramic membrane (Al_2_O_3_)	hollow fiber	13.0%	MEA (30%)		0.18 (Nm^3^/h)	90%		(27)
					0.5 (Nm^3^/h)	70%	7.8 × 10^–3^	
	hollow fiber	15.0%	water	160 (cm^3^/s)	50 (Ncm^3^/s)	75%		(36)
			DEA (2 M)	160 (cm^3^/s)	100 (Ncm^3^/s)	99%		
composite PTFE/PES	hollow fiber	20%	deionized water		100 (mL/min)		1.0 × 10^–4^	(37)
			NaOH (0.08 M)				1.8 × 10^–4^	
ceramic membrane (Al_2_O_3_/ZrO_2_)	tube	indoor air (CO_2_:380 ppm)	NaOH (2 M)	20 (mL/min)	300 (mL/min)	98%	7.9 × 10^–4^	this work

a PVDF, polyvinylidene fluoride; PTFE, polytetrafluoroethylene; PES, polyethersulfone; NaOH, sodium hydroxide; MEA, monoethanolamine; DEA, diethanolam.

### 4.3 Effect of Gas Flow Rate on CO_2_ Removal Efficiency

**Figure 5:**
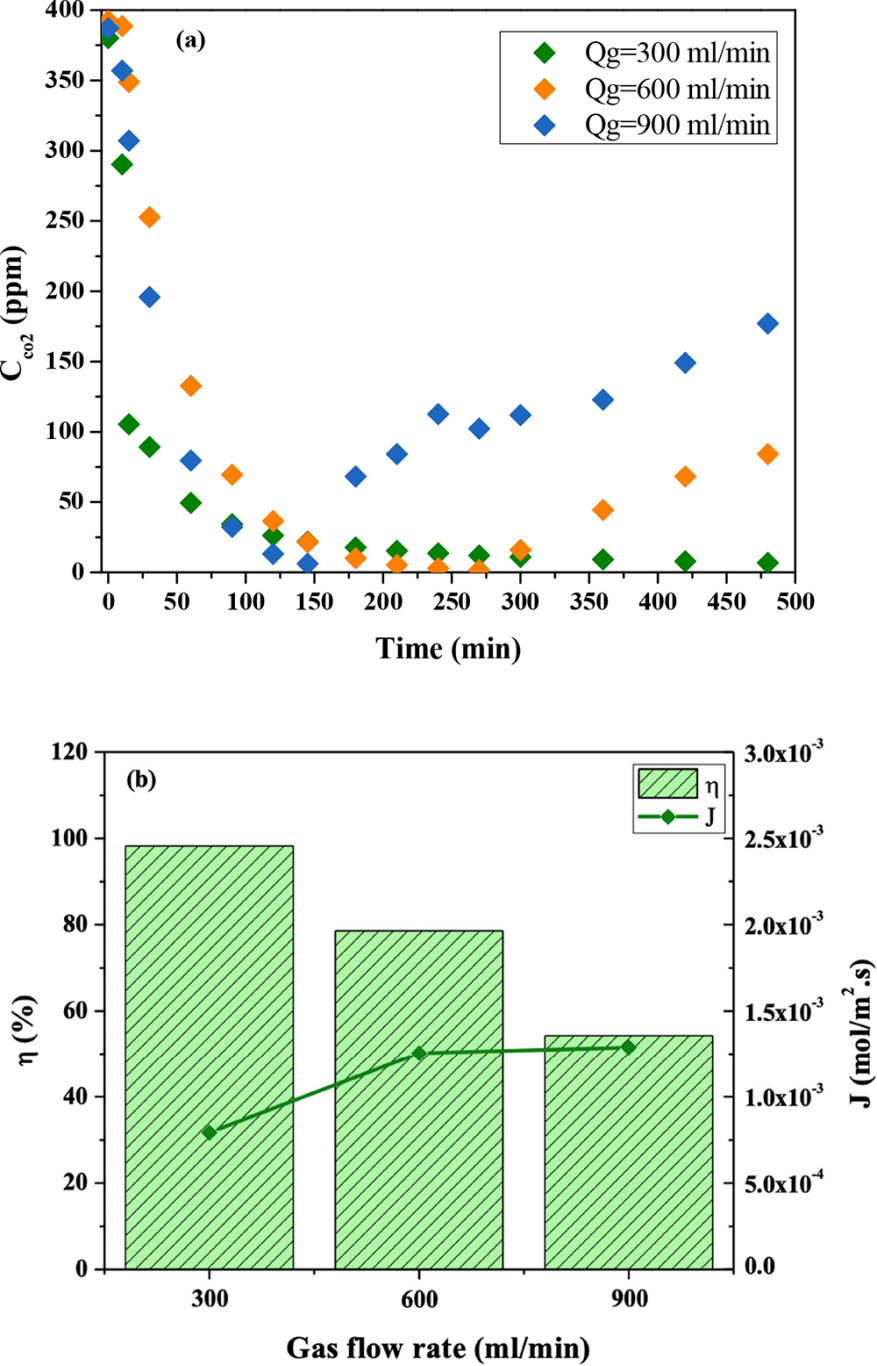
(a) Time-dependent variation of CO_2_ concentration in outlet gas flow at different gas flow rate (*Q*_g_), and (b) effects of gas flow rate (*Q*_g_) on CO_2_ removal (worked at *Q*_L_ = 20 mL/min and NaOH concentration = 2 M).

As shown in Figure 5a, an increase in *Q*_g_ to 900 mL/min caused CO_2_ levels to drop to 0 ppm after 145 min. However, as was observed, CO_2_ levels increased rapidly thereafter. At a *Q*_g_ of 600 mL/min, the saturation point was reached at 270 min, followed by a significant increase in CO_2_ concentration. This is due to the fact that the gas phase boundary layer becomes thinner as the feed gas rate increases, and the overall transfer coefficient increases until the gas resistance is ignored.^30^

Mass transfer flux is another important factor in understanding CO_2_ removal with the MC system. The mass transfer flux values calculated using eq 2 at different *Q*_g_ are shown in Figure 5b. As observed, a high *Q*_g_ increases the gas pressure on the outer surface of the tubular membrane, which forces the absorbent through the membrane pores, reducing the wettability of the membrane and increasing the CO_2_ flux.^29^ By preventing the wetting of the membrane pores, the mass transfer resistance of the membrane is reduced, so that the gas can more easily reach the liquid absorbent side. As can be seen in Figure 5b, when *Q*_g_ was increased from 300 to 600 mL/min, the CO_2_ mass transfer flux increased, accompanied by a decrease in CO_2_ removal efficiency (η). This was observed while the flux remained unchanged when *Q*_g_ increased to 900 mL/min. At high *Q*_g_, in other words, with the decrease of *Q*_L_/*Q*_g_, the residence time of CO_2_ decreased, which is unfavorable for CO_2_ absorption. This shows that the negative effect of the short residence time outweighs the positive effect of the high CO_2_ mass transfer rate in this case. At the low *Q*_g_ used in this study, a continuous decrease in CO_2_ concentration was observed until the end of the experiment (i.e., after 8 h).

### 4.4 Effect of Different NaOH Concentrations on CO_2_ Removal Efficiency

**Figure 6:**
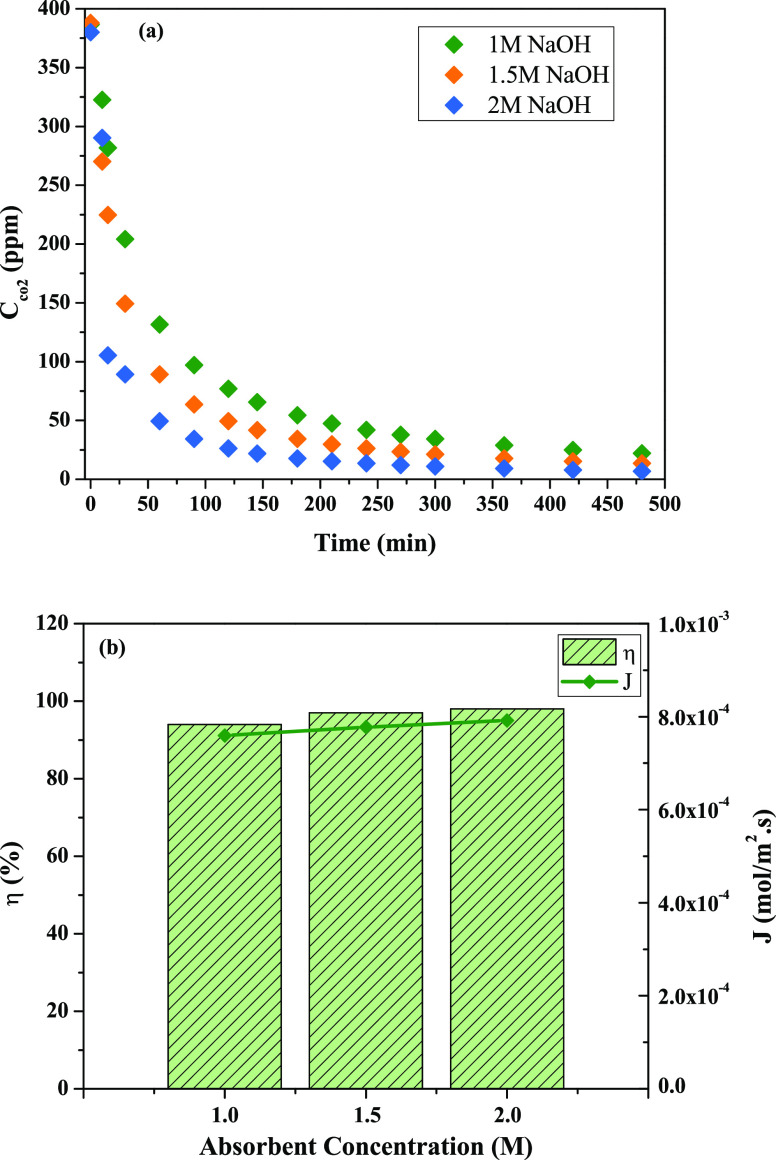
(a) Time-dependent variation of the CO_2_ concentration at different NaOH solution concentrations, and (b) effects of NaOH concentrations on CO_2_ flux and removal (*Q*_g_ = 300 mL/min and *Q*_L_ = 20 mL/min).

In this step, experiments were conducted with different NaOH concentrations at optimum *Q*_g_ (300 mL/min) and *Q*_L_ (20 mL/min) determined in this study. The values of NaOH concentration used in the literature were investigated, and the effects of 1, 1.5, and 2 M NaOH were studied.^24^Figure 6a shows that the CO_2_ concentration in the system operated with 1 and 1.5 M NaOH solutions is very close. When using 2 M NaOH solution, the CO_2_ concentration decreased even faster.

Various studies have used different absorbent solutions in MC systems, such as water, NaOH, amine-based absorbents, etc.^31,36^ It was shown that absorption in the presence of a chemical absorbent is determined by the chemical reaction between the absorbent and CO_2_. An increase in the absorbent concentration leads to an increase in the mass transfer of CO_2_ due to the increase in the reaction rate.^36^ In the current study, NaOH was used as the absorbent. The reaction between CO_2_ and [OH] increases the concentration gradient because the CO_2_ transferred to the liquid phase is rapidly consumed.^37^ A high NaOH concentration provides a high [OH], a higher reaction rate, and mass transfer as shown in eqs 8–10. Membrane channels have low mass transfer resistance and can increase the mass transfer coefficient of channels with increasing NaOH concentrations.^30^ The results of the current study were in line with other studies in which increasing the absorbance concentration from 1 to 2 M provided almost total removal of CO_2_:^31,36,37^
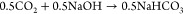
8
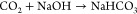
9
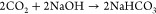
10

As can be seen from Figure 6b, the *J*_CO_2__ values at 1.0, 1.5, and 2.0 M NaOH were 7.59 × 10^–4^, 7.78 × 10^–4^, and 7.92 × 10^–4^ mol/m^2^ s, respectively. These results indicate that changing the concentration of the absorbing solution has no noticeable effect on the *J*_CO_2__ values. As a result of these experiments, the optimum conditions for CO_2_ removal were found to be 2 M NaOH solution, 300 mL/min *Q*_g_, and 20 mL/min *Q*_L_.

### 4.5 Management of the Reusability of TCM

**Figure 7:**
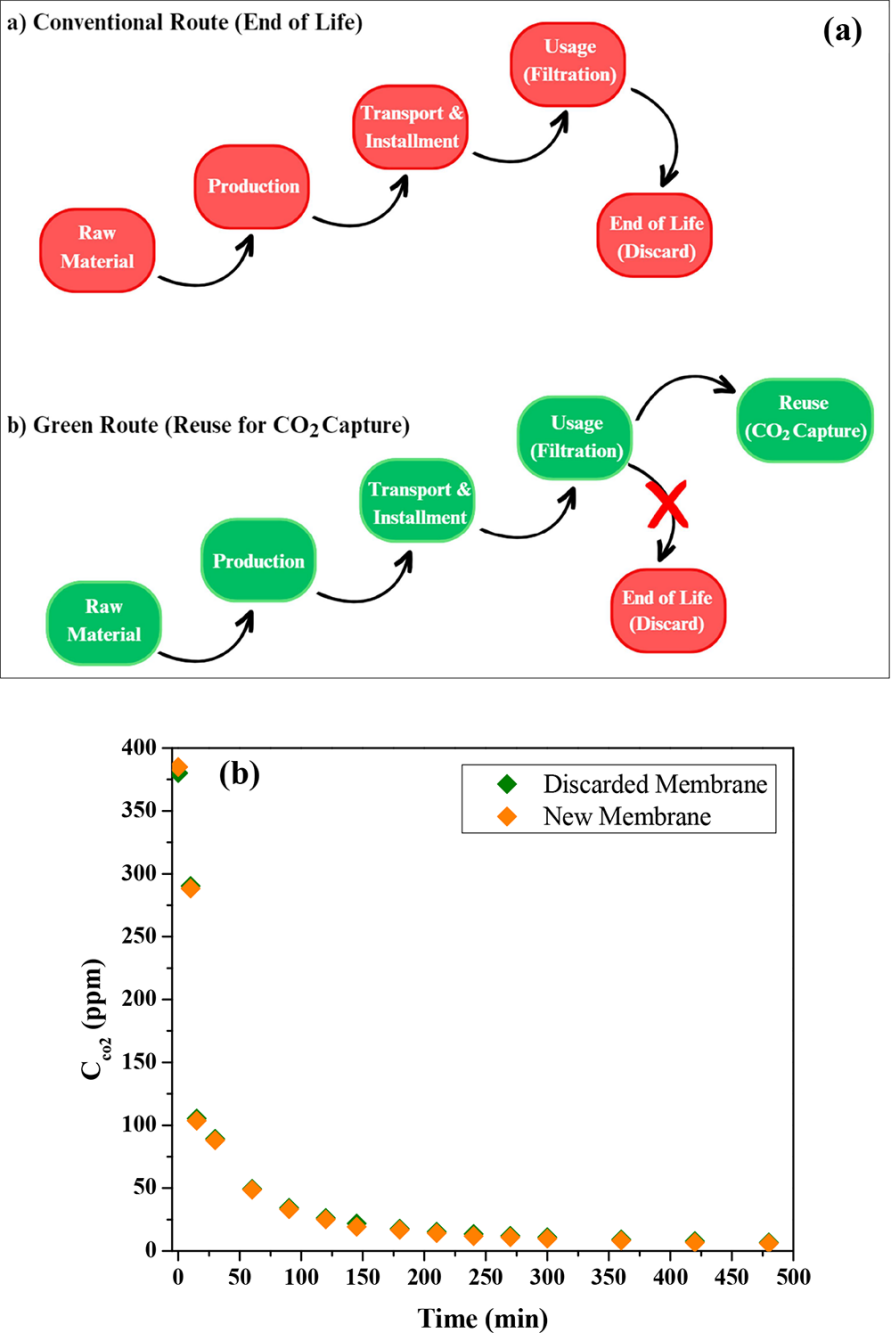
(a) Comparison of the conventional and green routes (sustainable life cycle approach) for discarded ceramic membranes, and (b) comparison figure between discarded and new membranes at optimum conditions.

According to the main principles of waste management, reuse and recycling of membranes should be considered an environmental measure to improve the sustainability of membrane technology and minimize the environmental impact. The main objective of this study was to recycle and convert discarded ceramic membranes into a laboratory-scale MC for CO_2_ capture and removal. The CO_2_ removal parameters obtained showed their validity and suitability for the MC process. Their operating parameters were found to be very similar to those of the applications. In addition, this study aimed to draw attention to the reuse of ceramic membranes in alternative applications. Figure 7a illustrates the differences between the conventional and sustainable routes of the life cycle of ceramic membranes. The sustainable pathway suggests using ceramic membranes in other applications and avoiding the generation of waste. Furthermore, a wise choice of new applications of ceramic membranes could serve to control GHG emissions in a more sustainable way. Looking at how ceramic waste is generally evaluated in the literature, it can be seen that studies suggest the reuse of ceramic waste from construction sites or from the construction industry as a raw material substitute for natural aggregates in structural concrete.^38^ However, our results showed that the discarded ceramic membranes still have promising potential in other areas of environmental technology. Figure 7b shows that at the end of their lifetime, the ceramic membranes still have more or less the same economic value as before their use. In this way, a material that has very high production costs and high technical importance is appropriately valued. This study offers an alternative reuse in which the ceramic membranes are not considered a waste material, but an important element in another process. The fact that used or discarded ceramic membranes can be used for gas separation in MC systems with chemical absorption solutions that polymeric membranes cannot withstand will ensure that these membranes can be used for a very long time. This approach therefore not only offers a new reuse potential for discarded ceramic membranes but also reduces the environmental impact associated with their disposal and provides additional environmental value through their contribution to CO_2_ removal.

MC systems that combine the absorption process with membranes are an emerging membrane technology in the literature with promising potential. However, optimized real-scale applications are not yet available. This could be due to uncertainties in the long-term stability of the whole system, wettability, thermal resistance, and the effects of other gases that could interrupt the whole process or affect the efficiency of the MC system.

## 5 Conclusions

Membranes are widely used in water/wastewater treatment and resource recovery. However, after their effective use, the waste load is very high, considering that the average lifetime of membranes is short and they need to be replaced quickly. To implement a more sustainable approach to membrane technology in relation to the management of used and discarded membranes, the recycling and reuse of these membranes should be considered. The introduction of new approaches in membrane science and technology to implement a circular economy action for membrane reuse is of great importance in the near future. The present study can be considered a preliminary study for the possible reuse of ceramic membranes that have exceeded their service life in MC systems for CO_2_ removal from indoor air. The aim of the present study was to simulate the potential scenario of discarded ceramic membranes in an alternative process. The results of this study showed that under optimum conditions of 2 M NaOH, *Q*_L_ (20 mL/min), and *Q*_g_ (300 mL/min), 98% of CO_2_ could be captured, which lasted for 8 h of the experimental observation period. Furthermore, the results obtained in this study suggest that discarded ceramic membranes should be re-evaluated and promoted for further novel applications in the fields of sustainability and global warming. As a recommendation for further work in the future, similar studies should investigate the optimization of MTS concentration, immersing time, and morphological changes.
